# Drowning Mortality and Morbidity Rates in Children and Adolescents 0-19yrs: A Population-Based Study in Queensland, Australia

**DOI:** 10.1371/journal.pone.0117948

**Published:** 2015-02-25

**Authors:** Belinda A. Wallis, Kerrianne Watt, Richard C. Franklin, James W. Nixon, Roy M. Kimble

**Affiliations:** 1 Centre for Children’s Burns & Trauma Research, Queensland Children’s Medical Research Institute, The University of Queensland, Brisbane, Australia; 2 Paediatric Burns and Trauma Network, Royal Children’s Hospital, Brisbane, Australia; 3 College of Public Health, Medical and Veterinary Sciences, James Cook University, Townsville, Australia; 4 Royal Life Saving Society Australia, Sydney, Australia; University of Bremen, GERMANY

## Abstract

**Objective:**

To redress the lack of Queensland population incidence mortality and morbidity data associated with drowning in those aged 0-19yrs, and to understand survival and patient care.

**Design, Setting and Participants:**

Retrospective population-based study used data linkage to capture both fatal and non-fatal drowning cases (N = 1299) among children aged 0-19years in Queensland, from 2002-2008 inclusive. Patient data were accessed from pre-hospital, emergency department, hospital admission and death data, and linked manually to collate data across the continuum of care.

**Main Outcome Measures:**

Incidence rates were calculated separately by age group and gender for events resulting in death, hospital admission, and non-admission. Trends over time were analysed.

**Results:**

Drowning death to survival ratio was 1:10, and two out of three of those who survived were admitted to hospital. Incidence rates for fatal and non-fatal drowning increased over time, primarily due to an increase in non-fatal drowning. There were non-significant reductions in fatal and admission rates. Rates for non-fatal drowning that did not result in hospitalisation more than doubled over the seven years. Children aged 5-9yrs and 10-14yrs incurred the lowest incidence rates 6.38 and 4.62 (expressed as per 100,000), and the highest rates were among children aged 0-4yrs (all drowning events 43.90; fatal 4.04; non-fatal 39.85–comprising admission 26.69 and non-admission 13.16). Males were over-represented in all age groups except 10-14yrs. Total male drowning events increased 44% over the seven years (P<0.001).

**Conclusion:**

This state-wide data collection has revealed previously unknown incidence and survival ratios. Increased trends in drowning survival rates may be viewed as both positive and challenging for drowning prevention and the health system. Males are over-represented, and although infants and toddlers did not have increased fatality rates, they had the greatest drowning burden demonstrating the need for continued drowning prevention efforts.

## Introduction

Drowning is preventable, yet in most countries drowning ranks among the top three causes of injury death.[1,2] The estimated drowning global mortality rate of 6.8/100,000 translates to approximately 400,000 deaths annually.[3] Almost half of those deaths worldwide are children and adolescents aged 0–19yrs, accounting for 175,000 lives lost in a single year.[1] Deaths from drowning among children and adolescents is second only to road traffic trauma as a principal cause of unintentional injury death.[1] Furthermore, the magnitude is greater than these figures reveal, because global numbers do not include drowning due to floods, cataclysms, transport incidents and intentional drowning.[2]

Drowning is an injury with particular etiological patterns that change according to age group, aquatic setting and activity.[4] Many studies focus on either mortality or hospital admissions, with very few publishing combined figures. Reporting across these various categories makes a uniform measure of drowning difficult to define, and consequently it is difficult to compare findings across studies or conduct meta-analyses in order to implement preventative strategies. Additionally, countries categorised by income such as high income countries (HIC) or low and middle income countries (LMIC) have broadly varying death rates.[1] Worldwide, the greatest burden for drowning fatalities occurs in the youngest children and global rates for 0–4yrs at 18.9/100,000 and for 5–14yrs at 9.5/100,000 confirm this.[5] Among children aged less than 15yrs in the Western Pacific region (which includes Australia), fatal rates range from 1.2–13.9/100,000. The rates for the most vulnerable children aged 1–4yrs varied from 2.0–10.2/100,000.[6]

The state of Queensland has the second highest number of drowning deaths comprising 22% of drowning deaths Australia-wide.[4,7] The most recently available data show downward trends for Australian drowning mortality and hospitalisation from 1999–00 to 2003–04. However, the drowning mortality rate in Queensland (IR = 2.2/100,000) is significantly higher than for Australia (IR = 1.9/100,000), as is the hospitalisation rate (4.2/100,000 Queensland vs 3.0/100,000 Australia).[8] The National Water Safety Strategy, the Annual Report of Deaths of Children and Young People, and state government departments of Health, and Local Government have all indicated that prevention of drowning is a high priority,[9,10] with drowning fatalities among children 0–17yrs increasing over the five years up to 2008–09.[10] Furthermore, state-wide non-fatal data on the outcomes of drowning events are not collected, so the true morbidity burden from this injury on particular sectors of the population is unknown.

A population-based study of deaths and survivors of drowning episodes has not previously been conducted across Queensland, nor across the age spectrum 0–19 yrs. It is at least three decades since Pearn & Nixon,[11] and Pitt & Balanda[12] conducted their review of 0–14yr-old child drowning incidents in the City of Brisbane Drowning Study. In these studies the rates for fatal and non-fatal freshwater drowning for ages 0–14yrs were reported as more than doubling from 10.4/100,000 to 26.8/100,000 in the decade 1976 to 1986.[13,14] Rates for 0–4yrs increased from 26.6 to 70.2, and for all of Queensland were reported as 32.55/100,000 in 1997.[15] It is therefore timely for a detailed review that encompasses children of all ages, including the lesser-studied adolescent group who to date have been overlooked in Queensland studies. These data will assist in understanding of drowning incidents for prevention efforts, and will allow comparisons across national and international boundaries.

This study redresses the lack of a Queensland population-based study in those aged up to 20yrs, and presents analyses of fatal as well as non-fatal drowning data collected using data linkage. This method achieved the best possible case capture to report both fatal and non-fatal cases of drowning as defined internationally.[16,17] Data from across the patient journey provides vital detailed information of the drowning environment and patient care prior to, during, and after the incident.

## Methods

In this population-based study, data linkage was used to ascertain cases of fatal and non-fatal drowning events among children and young people aged 0–19yrs inclusive, residing in Queensland between January 2002 and December 2008. Data were accessed from multiple portals, including pre-hospital, emergency, hospital admission and death data, using the most appropriate data extraction criteria (Table 1).

**Table 1:** Data custodians, scope and extraction criteria—7 year review of Drowning in children and Adolescents in Queensland 2002–2008.

Pre-hospital, Hospital Emergency and Hospital Admission	Data Scope	Criteria for data extraction	Data coverage
Queensland Ambulance Service (QAS) https://ambulance.qld.gov.au/research.html (Accessed Oct 2014)	Fatal and Non-fatal, Pre-hospital, Queensland	Case nature = drowning; eARF = 0–19yrs; AIMS = all ages†	Information regarding all patients attended by QAS; data includes clinical information (vital signs) treatment administered by QAS or bystanders (e.g. CPR), facility and transfer details, and details of incident (location, events leading to)
Queensland Injury Surveillance Unit (QISU) http://www.qisu.org.au/ModCoreFrontEnd/index.asp?pageid=109 (Accessed Oct 2014)	Fatal and Non-fatal, Emergency, Admissions, 25% of Queensland (a)	Nature of main injury = drowning and submersion; age = 0–19yrs	Emergency presentations to 20 hospitals (a)
Emergency Department Information System (EDIS), Hospital Access Unit, Queensland Health http://www.health.qld.gov.au/research-reports/data-requests/default.asp (Accessed Oct 2014)	Fatal and Non-fatal, Emergency, 63% of Queensland (b)	ICD10 T751 Drowning and non-fatal submersion and ICD10 W65-W74; age = 0–19yrs	**Emergency presentations** to 101 hospitals with average 18,200 patients per year(b)
Surgical and Retrieval Team (SATR), Hospital Access Unit, Queensland Health, http://www.health.qld.gov.au/research-reports/data-requests/default.asp (Accessed Oct 2014)		ICD10 T751 Drowning and non-fatal submersion and ICD10 W65-W74; age = 0–19yrs	
Queensland Health Admitted Patients Data collection (QHAPDC), Health Statistics Centre, http://www.health.qld.gov.au/research-reports/data/	Fatal and Non-fatal, Admissions, Queensland	Principle Diagnosis/Other Diagnosis = ICD10 T75.1 Drowning and non-fatal submersion; age-0 = 19yrs	**Admitted patients** from all public and private hospitals in Queensland
Mater Health Services (MHS) (children and adults), Business Practice Improvement, http://www.mater.org.au/Home/Services, (Accessed Oct 2014)	Fatal and Non-fatal, Emergency, Admissions, Queensland (c)	Diagnosis description = Injury or Trauma including non-venomous bites drowning or near drowning 994.1; or Diagnosis = ICD10 T751; Drowning and non-fatal submersion); age = 0–19yrs	**Emergency presentations** to Mater Children’s and Adults Hospitals
**Fatal drowning data**	**Data Scope**	**Criteria for data extraction**	**Data coverage**
National Coronial Information System (NCIS), http://www.ncis.org.au/how-to-access-data-on-the-ncis/, (Accessed Oct 2014)	Fatal, Queensland 0–19yrs	Queensland jurisdiction; “Threat to breathing and downing and immersion” and string searches “drown*” and “immersion” and various truncations; year of death 2002–2008 inclusive	Includes coroners’ findings, police investigation reports and toxicology
Commission for Children and Young People and Child Guardian Child Death Review Unit (CCYPCG)‡, http://www.qfcc.qld.gov.au/contact-us info@qfcc.qld.gov.au	Fatal, Queensland, 0–17yrs only	Death registered as drowning; 0–17yrs; Year of death 2002–2008 inclusive	**Fatality data up to 17yrs**: Includes coroners’ data, death registration, police report and other information specific to mechanism
Royal Life Saving Society of Australia (RLSSA), http://www.royallifesaving.com.au/contact-us	Fatal, Queensland, 0–19yrs	Drowning data for Queensland year of death 2002–2008 inclusive, 0–19yrs	**Fatality data:** Includes coroners’ data, ABS data; member reports, details of rescues, and media reports

†eARF (Electronic Ambulance Report Form 2007–2008) and AIMS (Ambulance Integrated Management System 2002–2006).

‡CCYPCG has transitioned to the Queensland Family and Child Commission (QFCC) from 1 July 2014.

Abbreviations: CPR—Cardiopulmonary Resuscitation; ICD10—International Classification of Diseases Tenth Revision; ABS—Australian Bureau of Statistics.

a. 20 hospitals contribute data to QISU which approximates 25% of the state.

b. In 2008, 150 hospitals in Queensland saw an average of 86,700 patients per year. There were 101/150 hospitals in Queensland who operated with an electronic data system seeing an average of 76,300 patients per year. 12% patients attend hospitals without electronic emergency department systems.

c. Mater data covers public and private hospitals in Brisbane and environs servicing approx.45,000 emergency department child presentations/year and 37,000 Adult presentations per year. Of the 5 hospitals 2 are children’s hospitals and 3 adult (two private and one public). Mater Children’s Hospital provided data for children 0–17yrs inclusive. Mater Adult Hospital provided data for adolescents 18 and 19yrs inclusive. Approximately 23% of child admissions are through emergency department, and 27% of adult admissions.

Identified data were linked manually for each unique drowning event over the time period to collate comprehensive detail across the continuum of care. Drowning is a relatively unusual event which generally requires a patient to be transported for medical intervention immediately after the event. Consequently any disparity in time/date of presentation, and facility, or any duplication of any of these variables was highly unlikely. Identified data collected included: name, address, date of birth, medical record number, sex, facility name, date of incident/presentation/transfer, and mode of arrival and discharge status. BW and KW and three research assistants (MT, CM and HW) were responsible for merging and linking the data using Excel and Filemaker. BW & KW scrutinised data for consensus as to a correct match on a case by case basis. Identifiers such as date of birth, address, medical record number, or sex were able to be linked where names were spelled differently. Few difficulties were encountered in linking and matching identified data with overlapping variables between datasets, and discrepancies between two entries with differing data (e.g. address and postcode) were resolved by allocating a hierarchy to databases. Each case did not necessarily appear in all datasets. A case was included if it appeared in any dataset, provided it met the search criteria and included age. Included cases were categorised as pre-hospital, emergency attendances or admissions if they were present in any of these databases, but were only included once. Transfers between hospital facilities were excluded. The final dataset comprised unique drowning events during the study period. Further detail on data linkage is available from the corresponding author on request and is the subject of a forthcoming paper.

This study used the internationally agreed terminology for the drowning process outcome being either fatal or non-fatal drowning.[16,17] The scope of the data collected was deliberately broad to capture as many cases of non-fatal drowning as possible. Respiratory impairment (which excludes water rescue) is not readily extracted without further data mining, and the authors have assumed that if medical attention was not sought that respiratory impairment was unlikely. The authors have no way of knowing how many cases go unreported. The terms “all drowning events” or “total events” are used when referring to combined figures for both fatal and non-fatal drowning.

Crude incidence rates (IRs) were calculated for drowning events for each calendar year using population data from the Australian Bureau of Statistics (ABS) and are quoted as per 100,000 population. Rates were calculated separately for all drowning events; fatal events; and non-fatal events (hospital admission and non-admission were also calculated separately); by age group and gender.

Even though data for every drowning event that occurred in Queensland were extracted, IRs were calculated for events among Queensland residents only, due to the lack of age-specific population data on non-Queensland residents (for the denominator). Trends over time were analysed by chi-square test for trend using Epi Info (7.0). Relative Risk and 95% Confidence Intervals (CI) were also calculated using IBM SPSS Statistics 22.

Ethics and approvals were sought and granted from Children’s Health Services District (Royal Children’s Hospital Human Research Ethics Committee HREC/09/QRCH/38; Royal Children’s Hospital Institutional Approval; University of Queensland Medical Research Ethics Committee #2009001463; Mater Health Services Human Research Ethics Committee #1446E; and National Coronial Information System #CF/07/13729 (2007–2010), #CF/10/25057 (2010–2013), #CF/13/19798 (2013–2016). Director General approval was granted for access to identified data without consent through Public Health Application, Queensland Health 16/3/2010 Ref RD002254. Custodian approvals were granted from Royal Life Saving Society Australia, Commission for Children and Young People and Child Guardian, Queensland Ambulance Service.

## Results

### Overall drowning 0–19 years

Between January 2002 and December 2008 there were 120 fatal and 1179 non-fatal drowning incidents (total = 1299) among 0–19yr-olds in Queensland. This equates to approximately three incidents per week. The ratio of fatal to non-fatal drowning events was 1:10, and for non-fatal events the ratio of hospital admission (n = 742) to non-admission (n = 437) was 1.7:1. Indigenous status was recorded for 80% of drowning incidents, and of those 7% of incidents involved children and adolescents who identified themselves as Aboriginal and/or Torres Strait Islander.

**Fig 1:**
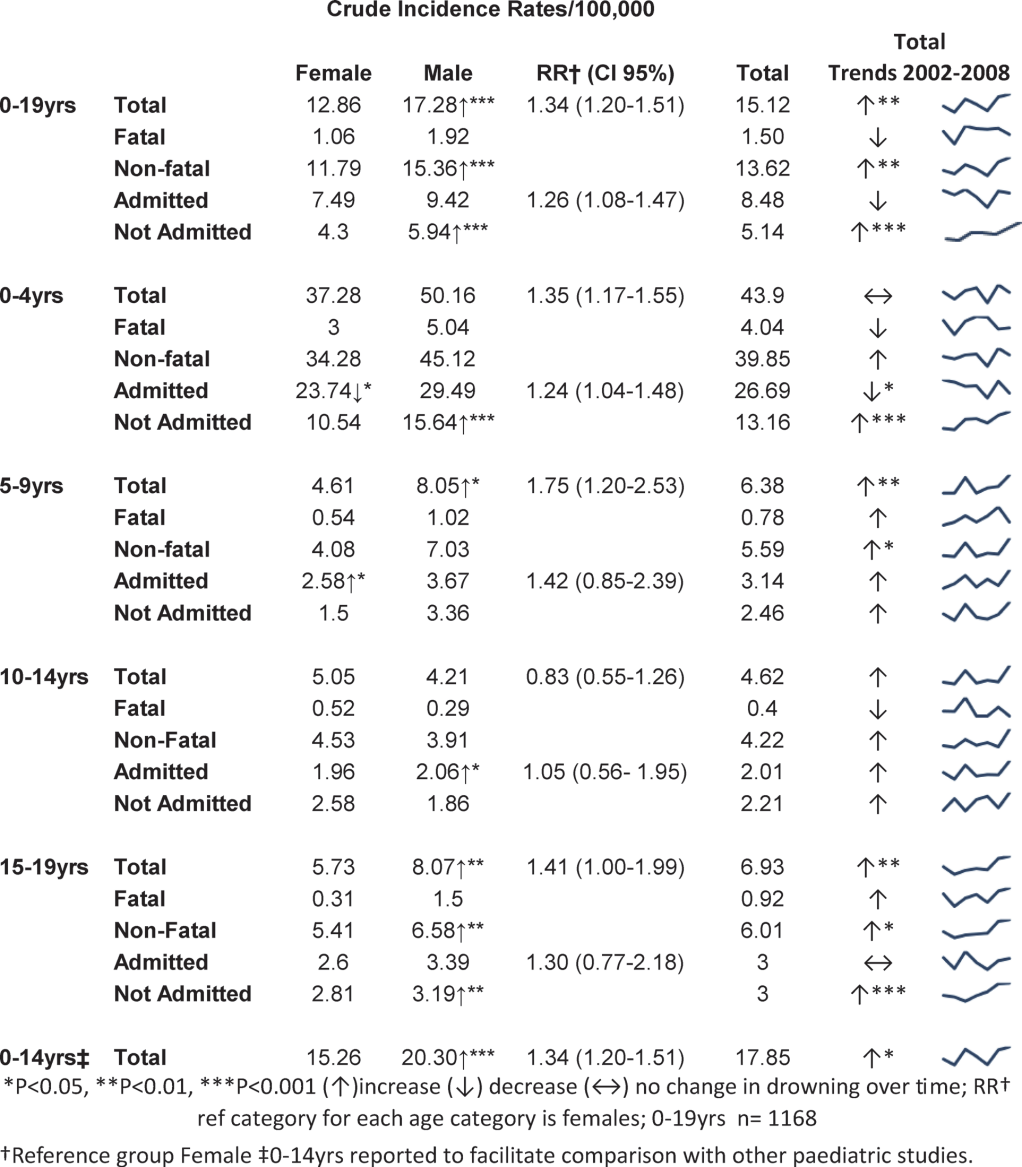
Incidence rates for all drowning, admitted, and not admitted drowning events by age group, gender and trends over time for 2002–2008.

The remaining analyses for IRs relate only to Queensland residents (n = 1168). There were 116 fatal and 1052 non-fatal events among 0–19yr old Queensland residents yielding an overall total drowning incidence rate of 15.12/100,000 (Fig. 1). Incidence rates (IRs) for all drowning events increased by 28% during the study period (X^2^ = 9.02; P<0.01) and during 2006–2008 rates increased by 44%. There was a non-significant reduction in fatal IRs over the seven years (X^2^ = 0.44; P = 0.509), and non-fatal IRs increased by 33% (X^2^ = 8.41; P<0.01).

**Fig 2:**
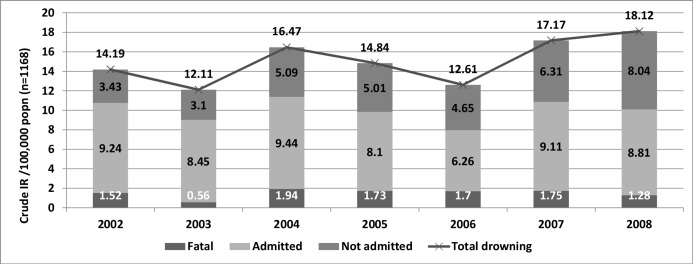
Drowning incidence rates in 0–19yr old Queensland residents by calendar year 2002–2008 stratified by severity.

Sixty two percent (n = 655) of children involved in non-fatal drowning events were admitted to hospital, and of those (n = 408) who were not admitted 84% attended the emergency department (ED) and the remaining 16% (65) received emergency pre-hospital care only. Fig. 2 shows the rates for non-fatal drowning. The rates for survivors who sought medical help but were not admitted to hospital increased significantly, more than doubling to the end of the study period by a factor of 2.34, (IR = 5.14/100,000; X^2^ = 30.48; P<0.001) while hospital admissions reduced slightly (IR = 8.48/100,000; X^2^ = 0.40; P = 0.530).

Males were involved in just over half (59%) of all drowning events (M:F 1.3) and were 1.3 times more likely than females to be involved in a drowning incident (RR = 1.34; 95%CI = 1.20–1.51) (Fig. 1). During the study period there were very small non-significant reductions in both male and female drowning deaths. The incidence of drowning events increased by 44% over the seven year study period among males (X^2^ = 10.90; P<0.001), and this was primarily due to a 55% increase in non-fatal events (X^2^ = 10.96; P<0.001). For females, rates of drowning resulting in death, admission (X^2^ = 0.499; P = 0.480) or non-admission (X^2^ = 3.251 P = 0.071) reduced slightly, but not significantly. In contrast, there was a significant rate increase in males (174%) in drowning events that did not result in admission (X^2^ = 24.64; P<0.001). Most commonly patients spent 24–48 hours (62%) in hospital before discharge. A stay of less than 24 hours (24%) was the second most common time period.

### Age group & sex

Fig. 1 shows the IRs for the duration of the study stratified by age group and gender, all drowning separately, trends over time and relative risk of drowning for males.

**0–4yrs**: The majority (70%) of drowning events and the highest incidence rates consistently occurred in this age group regardless of severity. There were no significant changes in fatal or non-fatal rates. Admission rates reduced (X^2^ = 0.4.4; P<0.05) and non-admission rates more than doubled (X^2^ = 17.73; P<0.001). Of the children and adolescents who were hospitalised for 24–48 hours or less than 24 hours, 82% and 69% were 0–4yrs olds.

Males in this age group incurred the highest incidence rates for the entire study (IR = 50.16) and the risk of young males being involved in a drowning event was 1.3 times greater than for females of the same age (RR = 1.35 95%CI = 1.17–1.55). Male non-admission incidence rates increased (X^2^ = 15.34; P<0.001) whereas female hospital admission rates decreased over the time (X^2^ = 4.72; P<0.05).

**5–9yrs**: Children 5–9yrs made up only 10% of total drowning incidents overall however rates almost trebled (X^2^ = 7.96; P<0.01) over the study period. Deaths were few in this age group (maximum six in 2007) and in four of the seven study years there were either no recorded male fatalities or female fatalities (data not shown). Increased events were mostly non-fatal (X^2^ = 5.83; P<0.05).

For this age group the highest incidence in total drowning events occurred in males (X^2^ = 4.86; P<0.05) who also had a greater risk of drowning than their female counterparts of the same age (RR = 1.75 95%CI = 1.20–2.53). Male total drowning rates increased over the time of the study by a factor of 3.14 (X^2^ = 4.86; P<0.05). Incidence of hospital admissions in females increased by a factor of 5.63 (X^2^ = 4.00; P<0.05).

**10–14yrs**: The lowest incidence rates of the entire study were in young adolescents who also had the lowest risk of drowning. Admission rates increased significantly over time among males (X^2^ = 3.99; P<0.05).

There were three years of the study where no fatalities were recorded at all, and a further two years where there were no male fatalities. This was the only age group where numbers of non-fatal incidents in males were fewer than females. (RR = 0.834; 95%CI = 0.55–1.26).

**15–19yrs:** Older adolescents were involved in 12% of incidents overall. Total drowning incidence rates almost trebled over the study (X^2^ = 6.96; P>0.01) primarily due to non-fatal events (X^2^ = 5.31; P<0.05). Although the rates for hospital admissions and non-admissions were similar, the non-admission rate increased by a factor of 2.3 (X^2^ = 11.12; P>0.001) over the study.

For male adolescents total drowning rates almost doubled (X^2^ = 7.06; P<0.01), and non-fatal and non-admission rates more than doubled (X^2^ = 6.13; P<0.01; X^2^ = 8.72; P<0.01) respectively. Mortality rates for females were the lowest rates in this age group; no fatalities were recorded for four years of the study. The risk of a male drowning was 1.4 times that of a female (95%CI = 1.00–1.99).

### Relative Risk by Age Group

**(Table 2) 0–4yrs:** Children under 4yrs were the most vulnerable to a drowning event, though the risk of them being involved in a non-fatal event was higher than a fatal event. Children aged 0–4yrs were seven times more likely to be involved in a drowning event than children aged 5–19yrs and an almost 10-fold risk of being admitted to hospital.

**Table 2:** Relative risk of drowning by age for total drowning and hospital admission, Queensland residents 2002–2008.

Age (yrs)	IR total drowning (n)	RR† drowning event compared with 5–19yrs (95%CI)	IR Admission (n)	RR† hospital admission compared with 5–19yrs (95%CI)	RR‡ non-fatal drowning compared with fatal drowning (95%CI)
**<1**	35.84 (134)	6.01 (4.93–7.34)	18.46 (69)	6.81 (5.14–9.04)	15.75 (7.71–32.18)
**1**	67.6 (250)	11.34 (9.64–13.33)	41.37 (153)	15.27 (12.23–19.07)	9.00 (5.95–13.60)
**2**	56.56 (209)	9.49 (7.99–11.26)	34.37 (127)	12.69 (10.05–16.02)	8.95 (5.70–14.05)
**3**	40.83 (151)	6.85 (5.66–8.29)	25.96 (96)	9.58 (7.44–12.34)	7.39 (4.52–12.08)
**4**	18.86 (70)	3.16 (2.45–4.09)	13.47 (50)	4.97 (3.62–6.83)	22.33 (7.03–71.02)
**0 to 4**	43.9 (814)	7.36 (6.50–8.34)	26.69 (495)	9.86 (8.24–11.78)	1.82 (1.78–1.86)
**5 to 19**	5.96 (350)	1	2.71 (159)	1	7.54 (5.44–10.44)

RR†—Relative Risk reference group 5–19yrs; RR‡—Relative Risk reference group Fatal (non-fatal is any drowning event—pre-hospital and admitted); IR—Incidence Rates quoted per 100,000 population per annum.

Compared with 5–19yr olds, the risk of a drowning event for the youngest children aged 0–4yrs peaked at age 1yr, and remained elevated until the age of 4yrs before it fell to a 3-fold risk. The incidence of admission to hospital following a non-fatal drowning event was almost ten times higher among children 0–4yrs than older children and adolescents aged 5–19yrs.

The most vulnerable age was 1yr with the highest incidence for all drowning events, admission to hospital, and a ten-fold risk of being involved in a fatal drowning event compared with 5–19yr olds (RR = 9.68 95%CI = 5.89–15.91). Children aged either <1yr or 4yrs had the highest risk of being involved in a non-fatal drowning compared with a fatal drowning. For each year of age less than 5yrs, the risk pattern for those admitted to hospital is the same as for children involved in any drowning event.

## Discussion

### Overview 0–19yrs

Drowning prevention requires an understanding of all drowning scenarios, however there is no one database in Queensland which allows analysis of drowning deaths and survivals. This study linked data from ten databases to form a picture of fatal and non-fatal drowning in Queensland and demonstrates that fatal drowning is only a small part of the drowning problem.

On average, there were three drowning episodes each week in Queensland, and for every child or adolescent fatality, ten others were rescued, potentially resuscitated and survived. Two out of three of those survivors were admitted to hospital. Trends over the time of the study indicate that fatal drowning decreased slightly, but not with the same magnitude that non-fatal drowning rates increased.

The survival ratio for Brisbane City was 1:1 in 1976 for children aged 0–15yrs[13,18] and 1:3 a decade later.[14] A ratio of 1:9 for Queensland[15] in the mid-1990s was thought to be under-estimated as only hospital admissions were included. In line with national figures[7] fatalities did reduce slightly over the study period however, the overall drowning rate increased, and of concern is that in the last three years of data collection, the rates increased 44%.

The over-representation of male involvement in our data is supported in national data[19,20] and is evident across all years of the study and all types of drowning with two minor exceptions (10–14yrs fatal and non-fatal) where female rates marginally exceeded males. Non-fatal rates for males increased 55% which is of concern. On the one hand it is positive that these increased events did not result in fatalities, but this does warrant further investigation as to whether increases were associated with better supervision, resuscitation response, medical management, or continuing or persistent morbidity.

A modest but non-significant reduction in admission rates over the seven years is also a positive finding, however, numbers of drowning survivors who sought medical assistance, but were not admitted more than doubled. This previously unknown burden of non-fatal drowning shows more than 62% of drowning survivors being admitted to hospital and most having stayed for one or two days. The impost on the health system has yet to be explored.

Total drowning events among Aboriginal and Torres Strait Islanders in Queensland has not been previously documented. Seven per cent of all drowning incidents involved children and adolescents self-identified as Aboriginal and/or Torres Strait Islander. This population is proportionately 6.5% of the Queensland population and is therefore not over-represented.[21]

**0–4yrs:** Although 0–4yr infant and toddler fatality rates decreased, this group still bore the greatest mortality and morbidity burden from drowning. Non-fatal rates were seven times that of children and adolescents aged 5–19yrs. While fatality rates are much lower than reported in 1973 (IR = 15.69)[15,22,23] it is concerning that drowning in this age group has not decreased significantly over the period of the study. Compared with national data, the drowning fatality rate for Queensland toddlers was more than double[24] (1.8 vs 4.04) and admission rates were 48% higher.[8]

Total drowning rates for toddlers have continued to increase. Rates in Brisbane City increased 164% the decade following 1975 from 26.62 to 70.2.[13,14] The Queensland rate of 43.9 for this study is 65% higher than in 1997(IR = 32.55).[15] Children aged one year remain the most vulnerable as earlier studies have shown,[11,12,13] however it is encouraging that children aged <1yr and those aged 4yrs had a reduced risk of being involved in a fatal event compared with a non-fatal. The high risk of drowning associated with the early developmental years needs to be examined in the context of access to water and supervision. Pool fencing as a drowning prevention strategy for young children has been shown to be effective[12,25] and was mandated in Queensland in 1991[26]. Regulatory control by individual local councils across the State led to inconsistencies in enforcement so it is difficult to say definitively that this has reduced fatality on its own, however as the only statutory intervention promoted since 1991, this is a positive sign toward reducing drowning in this age group. Continued action is required to reduce drowning in toddlers 0–4yrs[27], and analyses indicate a high need for vigilance and continued intervention as the risk of drowning and admission to hospital for young children compared with that of older children was almost 10-fold.

**5–19yrs**: Numbers were small for fatal and non-fatal drowning and events fluctuated yearly over the study. No fatalities were recorded for older adolescents over several years of the study. Low fatality rates and reduced admission rates in older adolescents must be weighed against significant increases in non-fatal drowning and non-admissions as an impost on the health system. International data show similar patterns of drowning rates by age group, however, rates for Queensland are more than double those of the US.[28]

Further investigation is required to determine why adolescents 10–14yrs had the least drowning incidents, hospital admissions and non-admissions. All Queensland children are taught to swim at school from about age six, and this along with some level of supervision for this age group may account for the lower rates in children 5–14yrs. Also further investigation is warranted for the 10–14yr group where females had higher drowning rates than males (M:F 1:1.2). The male predominance was found in other Australian studies[27,29] and indicates a specific injury prevention need. The low rates in 10–14yrs drowning are reflected in similar patterns in Australian fatal drowning[4] and comparable rates of hospitalisation (3.0 for 15–24yrs vs 2.7 15–19yrs).[8]

### Data scope

In this study as many databases as possible were accessed for case capture, and we believe this is a sound foundation for a population-based study. This is the first study on drowning to comprehensively link datasets across the continuum of care (pre-hospital to fatality), to allow mapping of the patient journey for a drowning event. The inherent value of this approach is evidenced by the previously unexplored magnitude of non-fatal drowning which proved larger than expected, and the impact on health services is therefore apparent. Including morbidity data has enhanced the quality of data in relation to statistical analyses and age groups most at risk, and will inform injury prevention strategies.

The current drowning definition [16, 17] encompasses the fact that drowning can result in either death or survival from respiratory impairment. Further detailed data mining would be required to determine if respiratory impairment (which excludes water rescue) had occurred. These data would support an investigation to determine if this is achievable using the definition in its current form. For this paper, drowning survival where medical attention was sought was an attempt to exclude those who did not suffer respiratory impairment. Sixteen percent of non-admitted patients were treated by paramedics (pre-hospital) and were not transported to hospital, and there are a number (at this point unknown) who presented to the emergency department for precautionary care and who potentially may not have suffered respiratory impairment. Limitations in this study relate to the time involved in accessing and linking data, and the associated privacy issues which unnecessarily dates the data. Real time data collection inherently has a lag as Coroners can take several years to definitively confirm cause of death and close cases. Mortality figures available through the Australian Bureau of Statistics are underestimated for the same reason.[24] As data linkage continues to improve in Queensland it is hoped that future studies will allow for more effective and timely linked data.

### What this study adds

- An understanding of all drowning events that require care in Queensland for children 0–19yrs is gained by using data linkage (previously not undertaken for drowning).
- Previously unreported incidence of fatal and non-fatal drowning (IR = 15.12/100,000 pa), survival ratio 1:10 and ratio of hospital admission compared with non-admission 1:2.
- Overall there was a significant increase in 0–19yrs non-fatal drowning. Fatalities and admissions reduced however, not with the same magnitude.
- The rate of drowning survivors who sought medical assistance, but were not admitted to hospital, more than doubled.
- The highest incidence rates occurred in the 0–4yrs age group. This group recorded the highest rates for fatality, survival and admission and are higher than national data. Survivals and non-admissions increased, while fatalities and admissions to hospital decreased over the study period.
- Children aged 10–14yrs had the lowest incidence rates for all drowning events.
- Males were over-represented in all age groups except 10–14yrs.
- Significant increases were observed in male non-fatal drowning episodes presenting to hospital.

### Prevention implications

Most gains for prevention efforts should focus on:

- Children 0–4yrs; access to water and supervision, with particular attention on critical ages 1–3yrs.
- The reasons children and adolescents 10–14yrs had the lowest rates and the over-representation of males in all age groups except 10–14yrs.
- Whether there is any association between resuscitation response, medical management, or continuing morbidity with increased survival rates.

## Conclusion

For every child or adolescent drowning fatality in Queensland, ten others were rescued, revived and survived. Two out of three of those survivors were admitted to hospital. Queensland appears to have made gains in preventing fatal drowning, though not with the same magnitude as the increased trends in non-fatal events indicated. Admissions to hospital as a proxy for severity indicated that of the increased numbers of survivors who presented to hospital for treatment, the numbers who were not admitted more than doubled over the seven years. Males were a large part of the rises in rates. Such increased events should be viewed as both positive and challenging for drowning prevention, warranting additional investigation to explore whether survival is associated with resuscitation response, medical management, improved parental supervision, and continuing or persistent morbidity.

Prevention strategies should continue to target 0–4yr-olds with respect to access to water and supervision, particularly those aged 1–3yrs as this group had the highest fatality, survival and admission rates and are higher than national data. To arrest increased drowning requires closer scrutiny of those who survive drowning events for insights as to why children and adolescents 10–14yrs had the lowest drowning rates and why males are over-represented in all age groups except 10–14yr olds.
